# The Rose Croix

**Published:** 1907-08

**Authors:** 


**The Rose Croix.** A Novel. By **David Tod Gilliam,** M.D. Illustrated by **Ted Ireland.** The Saalfield Publishing Co. 1906.

The purely literary output of medical men has been added to by the publication of The Rose Croix by Dr. Gilliam, who has written a story which is intensely interesting and thoroughly absorbing. The delicacy of construction and the lack of apparent effort on the part of the author gives the book a distinction which is relatively rare in the fiction of men whose lives have been run in scientific grooves.

There is a delicacy and a charm in the pages which one finds immeasurably aggreeable and the tale is well worth the reading. It is told in simple fashion without any attempt at pretty writing, and in that lies the success of fictional authorship. The story is well sustained and the plot admirably brought through to the end of the tale. There is none of that fish-eyed fol-de-rol begot of some of the fashion plate novelists, and the dramatic incidents are rounded out delightfully without padding or windy wanderings. There's a medical interest in the story and there is a doctor, and he's the sort of doctor one will like to read about. The book is thoroughly interesting and enjoyable.

